# Discovering Words in Fluent Speech: The Contribution of Two Kinds of Statistical Information

**DOI:** 10.3389/fpsyg.2012.00590

**Published:** 2013-01-17

**Authors:** Erik D. Thiessen, Lucy C. Erickson

**Affiliations:** ^1^Department of Psychology, Carnegie Mellon UniversityPittsburgh, PA, USA

**Keywords:** statistical learning, word segmentation, lexical stress, infant language, phonology

## Abstract

To efficiently segment fluent speech, infants must discover the predominant phonological form of words in the native language. In English, for example, content words typically begin with a stressed syllable. To discover this regularity, infants need to identify a set of words. We propose that statistical learning plays two roles in this process. First, it provides a cue that allows infants to segment words from fluent speech, even without language-specific phonological knowledge. Second, once infants have identified a set of lexical forms, they can learn from the distribution of acoustic features across those word forms. The current experiments demonstrate both processes are available to 5-month-old infants. This demonstration of sensitivity to statistical structure in speech, weighted more heavily than phonological cues to segmentation at an early age, is consistent with theoretical accounts that claim statistical learning plays a role in helping infants to adapt to the structure of their native language from very early in life.

## Introduction

The ability to segment words from fluent speech is taken for granted by adults, but it represents a major accomplishment for infants. Unlike the white spaces between words on the written page, pauses do not consistently mark word boundaries in fluent speech. This is not troublesome for adults, who can identify word boundaries in large part due to their familiarity with the word forms in their native language (e.g., Nazzi et al., 2005; Norris and McQueen, 2008). Infants, though, begin the task of word segmentation unable to take advantage of familiar word forms. The challenge faced by infants is comparable to the task faced by adults attempting to identify words spoken in a foreign language. Nevertheless, infants succeed in this task before they have amassed a large lexicon of familiar word forms (e.g., Jusczyk and Aslin, 1995; Bortfeld et al., 2005). Two cues have been suggested to play a role in infants’ earliest ability to segment words from fluent speech: conditional statistical information, and information about the prosodic structure of words (Thiessen and Saffran, 2003). These cues are likely to work together in natural languages, but an open developmental question is which is available to infants earlier in development. In this series of experiments, we will examine the hypothesis that sensitivity to conditional structure is available from an earlier age, and that statistical learning helps infants discover the predominant prosodic structure of words in their native language.

There is no doubt that information about the prosodic structure of words plays a role in infants’ and adults’ word segmentation. The difference between stressed and unstressed syllables is perceptually available to infants from a young age (e.g., Jusczyk and Thompson, 1978; Weber et al., 2005). To the extent that stressed and unstressed syllable systematically occur in particular word positions, this distinction can serve as a cue to word boundaries. In English, for example, most bisyllabic content words follow a trochaic pattern: they begin with a stressed syllable, and are followed by an unstressed syllable (Cutler and Carter, 1987). English-learning infants prefer to listen to trochaic words over words with a weak-strong (iambic) pattern (Jusczyk et al., 1993). When exposed to a stream of syllables, English-learning infants and English-speaking adults treat the stressed syllables as word onsets (e.g., Cutler and Norris, 1988; Echols et al., 1997; Jusczyk et al., 1999). Importantly, though, not all languages show this trochaic predominance; lexical items in other languages may be predominantly iambic. Therefore, English-learners trochaic bias is likely acquired from experience with the language (Thiessen and Saffran, 2007).

By contrast, sensitivity to conditional statistical information does not require language-specific knowledge; it is a cue to word segmentation that is available cross-linguistically. This cue is relevant to word segmentation because sounds within a word are more likely to co-occur than sounds across word boundaries (Hayes and Clark, 1970). For example, *copter* is very likely to occur after *heli*; but many words could potentially occur after *helicopter*. Conditional statistics – such as transitional probability (e.g., Saffran et al., 1996) – reflect the likelihood of co-occurrence among elements of the input. A body of prior research indicates that both infants and adults are able to segment words from fluent speech on the basis of conditional statistical information. For example, artificial language experiments demonstrate that after exposure to a sequence of syllables, both infants and adults are able to distinguish between syllable groups with high conditional relations (i.e., words), and syllable groups with low conditional relations, such as groupings that occur across word boundaries (e.g., Aslin et al., 1998; Thiessen and Saffran, 2004).

A variety of different computational accounts have been proposed to explain sensitivity to conditional statistical information (for discussion, see Frank et al., 2010). The most successful of these models – clustering models – search for and store clusters of statistically coherent elements (e.g., Perruchet and Vinter, 1998; Orban et al., 2008). These models predict that after exposure to speech, participants should have extracted a set of candidate lexical items (e.g., Giroux and Rey, 2009). Research with both infants and adults is consistent with this prediction. For example, infants accept words from the synthesized speech in English utterances after exposure to a stream of synthesized speech (Saffran, 2001). Similarly, infants and adults learn labels for novel objects more easily when provided the opportunity to segment the labels from fluent speech (Graf Estes et al., 2007; Mirman et al., 2008).

In word segmentation tasks, for example, this means that exposure to fluent speech leads to learners extracting a set of candidate lexical items. Evidence that learners are extracting clusters of statistically coherent elements can be seen even for non-linguistic stimuli (e.g., Fiser and Aslin, 2005), suggesting that this extraction is a domain-general aspect of conditional statistical learning.

The fact that infants are capable of extracting and storing word forms is consistent with a statistical bootstrapping account of the development of word segmentation (Thiessen and Saffran, 2003). On this account, infants initially rely on language-universal cues – such as sensitivity to conditional statistical information – to segment words from fluent speech. Once they have identified and stored a set of word forms, they can identify the acoustic features that are consistent across them (e.g., Lew-Williams and Saffran, 2012). For example, if infants are exposed to a set of words in which stress consistently occurs on the first syllable, they will acquire a trochaic bias (Thiessen and Saffran, 2007). Once infants have discovered the acoustic features that are consistent in their proto-lexicon, they can use these features as cues to subsequent word segmentation (e.g., Johnson and Jusczyk, 2001).

This transition is from language-general to language-specific cues is thought to take place between 7 and 9 months. While 7-month-old infants rely on conditional statistical information to segment fluent speech, 9-month-old infants favor lexical stress, even if segmenting on the basis of stress contradicts conditional statistical information (Johnson and Jusczyk, 2001; Thiessen and Saffran, 2003). Recent research by Höhle et al. (2009), however, indicates that infants as young as 6 months are familiar with the predominant prosodic structure of words in their native language. Höhle et al. suggest that 6 months is below the age at which infants are able to segment words from fluent speech via conditional statistical cues. If so, the statistical bootstrapping account of infants’ prosodic learning is necessarily incorrect. Instead, this would suggest that language-specific prosodic cues may be the earliest cue infants use to segment words from fluent speech. Additionally, it would suggest that knowledge about the prosodic form of words arises from some source other than statistical learning, perhaps such as learning solely from words in isolation.

However, the claim that infants below 6 months are unable to segment speech on the basis of conditional statistical information may be incorrect. Evidence suggests that young infants and even neonates are sensitive to conditional statistical information (Kirkham et al., 2002; Teinonen et al., 2009; Kudo et al., 2011). Further, one prior experiment indicates that 5- to 6-month-old infants are able to segment fluent speech via conditional statistical information (Johnson and Tyler, 2010). In Experiment 1, we seek to provide additional evidence that infants are able to segment fluent speech below 6 months of age. Additionally, we will investigate whether infants at this young age prioritize conditional statistical information over lexical stress as a cue to word segmentation, consistent with the statistical bootstrapping account. In Experiment 2, we will investigate whether infants in this age range are capable of learning to use lexical stress as a cue to word segmentation.

## Experiment 1A

Within the word segmentation literature, it is commonly held that infants develop the ability to segment fluent speech by 7.5 months, citing a seminal study by Jusczyk and Aslin (1995). Before this age, researchers have asserted that infants lack the ability to extract words from fluent speech on the basis of statistical structure (e.g., Höhle and Weissenborn, 2003). Others have proposed that the ability to segment words from fluent speech via transitional probabilities is intact earlier (e.g., Thiessen and Saffran, 2003; Johnson and Tyler, 2010). Evidence from neuroimaging is consistent with this claim (e.g., Teinonen et al., 2009; Kudo et al., 2011). The goal of Experiment 1A was to provide further behavioral evidence that infants are capable of segmenting fluent speech via conditional statistical information below 6 months. To do so, we exposed 5-month-old infants to an artificial language in which the only cue to segmentation is higher conditional relations between syllables within words relative to syllables spanning word boundaries (part-words). If the ability to segment speech does not emerge until later than 7 months, these 5-month-old infants should not discriminate between words and part-words following familiarization with this fluent speech stream. However, if the ability to parse speech on the basis of statistical cues is intact at an earlier age, infants should discriminate between words and part-words.

### Materials and methods

#### Participants

Data were obtained from 10 participants between the ages of 5.0 and 5 months, 14 days (*M* = 5.10). To obtain data from 10 infants, it was necessary to run 13 infants. The additional three infants were excluded for crying during the testing session (1), average looking times of less than 3.0 s (1), or experimenter error (1). A sample size of 10 infants was used based on a power analysis using an effect size calculated from Thiessen and Saffran’s (2003) Experiment 3, of which this experiment is a replication with a younger age group.

#### Stimuli

The stimuli used in this experiment were identical to those used in Thiessen and Saffran’s (2003) Experiment 3. Infants were exposed to an artificial language containing four bisyllabic nonsense words: *diti*, *bugo*, *dapu*, and *dobi*. The language was synthesized using MacinTalk, and all syllables were produced with neutral stress. This language was constructed such that two of the words – *dapu* and *dobi* – occurred twice as often (90 times) as the other two words (*diti* and *bugo*, each of which occurred 45 times). This ensures that test item foils can be constructed that differ solely on their conditional probabilities, rather than on the frequency with which infants hear them (for discussion, see Aslin et al., 1998). Words occurred in a pseudo-random order, with the constraint that no word could follow itself. Syllable-to-syllable transitional probabilities were 100% within a word, and 33% at word boundaries. Because there were no pauses or other acoustic cues to word boundaries in this artificial language, the conditional probabilities (high within a word, low at boundaries) provided the only cue to word segmentation.

Two kinds of test items were created to assess infants’ ability to segment the language: words and part-words. The word test items were the infrequent words (*diti* and *bugo*) from the artificial language. Part-words were syllable conjunctions that occurred across the two more frequent words (*bida* and *pudo*). During the infants’ exposure to the artificial language, both words and part-words occurred equally often. Therefore, any difference in infants’ responses to these two kinds of test items is not due to the frequency with which they have heard the words or part-words.

#### Procedure

Infants were tested individually in a sound-attenuated testing room, seated on a caregiver’s lap 150 cm away from a 32′′ LCD monitor. An experiment outside the testing room observed the infant over closed-circuit video and recorded the duration of his or her gaze at the central monitor using the Habit X software (Cohen et al., 2004). To eliminate bias, parents were asked to wear headphones, and the experimenter was blind to the nature of the stimuli being presented. Two speakers situated next to the central LCD monitor were used to present the audio stimuli.

At the beginning of the experiment, the infants’ attention was attracted to the central LCD monitor by the presentation of a colorful Winnie the Pooh video, accompanied by an attention-getting phrase. Once the infant looked at the central monitor, the video was replaced by a static image of a checkerboard, and the artificial language began to play. The checkerboard remained on screen, and the language continued to play, for 2 min. At the end of this time, the attention-getting movie reappeared on the screen.

Once infants focused their gaze on the central monitor, the test phase began. During this phase, 12 test trials were presented. Six of these trials were word trials, and six were part-word trials. Each test item occurred on three trials during the testing phase. Test trials were presented in random order. A test trial began with the attention-getting movie playing on the central monitor drawing the infants’ gaze forward. When the observing experimenter pressed a key indicating that the infant had fixated, the monitor displayed a video of a looming green ball on a black background, while the speakers began to play the test item (either word or part-word) separated by 1.4 s pauses. For as long as the infant maintained their gaze on the central monitor, the test trial continued, up to a maximum of 20 s. When the infant looked away for more than two consecutive seconds, the test trial ended and the attention-getting video reappeared on the central monitor.

### Results

If infants were able to successfully segment the artificial language, they should respond differentially to word test trials than to part-word test trials (e.g., Saffran et al., 1996). While in principle, any group-level preference is indicative that infants are able to differentiate the items, the experiments most similar to this one have resulted in a novelty preference (e.g., Thiessen and Saffran, 2003, 2007). If infants in this experiment behave in the same way, they should look longer at test items that violate their expectations (i.e., part-words) than at test items that fit what they have learned (i.e., words).

**Figure 1:**
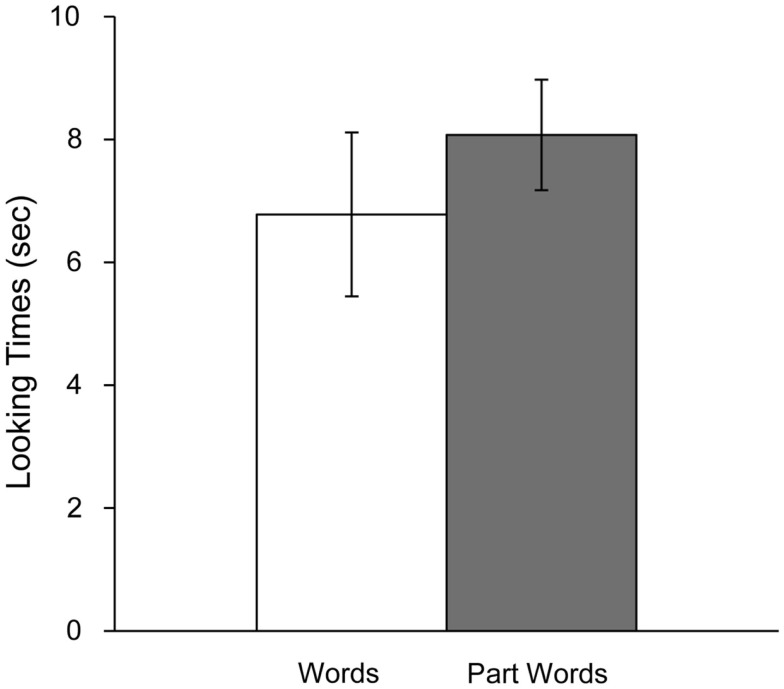
**Looking times to words and part-words in Experiment 1A**. Error bars indicate standard error.

The results were consistent with prior experiments using these stimuli. Infants in this experiment displayed a novelty preference, listening longer to part-words (*M* = 8.10 s, SE = 0.90) than words (*M* = 6.78 s, SE = 1.34; See Figure 1). A paired-samples *t-*test (all *t*-tests reported here and in subsequent experiments are two-tailed) revealed that the difference in listening times as a function of test item type was significant, *t*(9) = 2.609, *p* < 0.05. After familiarization, 5-month-old infants distinguished between words and part-words, indicating that they had succeeded in parsing the speech signal.

### Discussion

The fact that infants were able to segment the artificial language used in this experiment is inconsistent with the common assertion that speech segmentation does not begin until around 7 months of age (e.g., Jusczyk and Aslin, 1995; Höhle and Weissenborn, 2003). Instead, it is consistent with prior results indicating that infants are sensitive to conditional statistical information from a young age (Kirkham et al., 2002; Teinonen et al., 2009; Johnson and Tyler, 2010; Kudo et al., 2011). Indeed, to our knowledge the infants in this experiment are younger than any prior group of infants in a behavioral word segmentation experiment. The fact that they successfully segmented raises the possibility that word segmentation may begin at younger ages than previously thought in native language environments. Moreover, the 5-month-olds in this experiment are demonstrating sensitivity to conditional statistical information at a younger age than any prior experiment has found sensitivity to language-specific acoustic cues to segmentation, such as lexical stress patterns. As such, these results are consistent with the hypothesis that conditional statistical information is one of the first cues available to infants as they begin to discover word forms in speech.

## Experiment 1B

Experiment 1A demonstrated that 5-month-old infants are able to segment word forms from speech solely on the basis of conditional probability information. In Experiment 1B, we were interested in how infants of this age behave when statistical cues to word identity are placed in direct conflict with lexical stress, an acoustic cue thought to be very salient to infants (e.g., Gleitman et al., 1988; Echols and Newport, 1992). Much research attests to infants’ early sensitivity to prosodic information (e.g., Mehler et al., 1988) and preference that emerges at 9-months in English-exposed infants for trochaic words (consisting of a strong/weak pattern) over iambic words (weak/strong; Jusczyk et al., 1993). Additionally, 7.5-month-old infants in English-speaking environments are so reliant on lexical stress that they display a trochaic bias during segmentation, such that when exposed to passages containing the sequence “guiTAR#is,” they segment the trochaic sequence “TARis” from fluent speech even when it when occurs less frequently than the iambic sequence “guiTAR” (Jusczyk et al., 1999).

In the present experiment, we were interested in whether infants would extract units from familiarization on the basis of conditional information (i.e., extract syllable pairings characterized by high transitional probabilities) or on the basis of lexical stress cues (i.e., trochees following the dominant pattern of English). Based on the prior finding that 7-month-olds ignore stress cues, segmenting items on the basis of conditional information (Thiessen and Saffran, 2003), we predicted that 5-month-old infants in this study would also extract units according to this language-universal strategy rather than on lexical stress, which requires language-specific knowledge about words. If infants of this age segment statistical words rather than trochaic disyllables, this would provide strong support for the idea that conditional information is one powerful language-universal cue that could be recruited to acquire language-specific knowledge such as the preferred position of stressed syllables within word forms. In contrast, if these infants extract trochees from the speech stream, even when they are characterized by low transitional probabilities, this would be consistent with the early rhythmic segmentation hypothesis, proposed by Nazzi and colleagues (e.g., Nazzi and Ramus, 2003; Nazzi et al., 2006; Höhle et al., 2009; Mersad and Nazzi, 2011). According to this hypothesis, early segmentation is based on the rhythmic unit of the native language, which derives from infants’ early sensitivity to language rhythm.

### Materials and methods

#### Participants

Data were obtained from 20 participants between the ages of 5.0 and 5 months, 15 days (*M* = 5.9). Half of these infants were exposed to a trochaic artificial language, and half to an iambic artificial language. To obtain data from 20 infants, it was necessary to run 23 infants. The additional three infants were excluded (two from the trochaic condition, one from the iambic condition) for crying during the testing session. A sample size of 10 infants for each language was used based on a power analysis of Thiessen and Saffran’s Experiment 2, of which this experiment is a replication with a younger age group.

#### Stimuli

**Figure 2:**
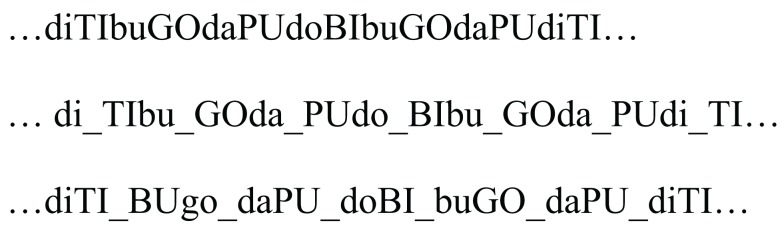
**Top: an excerpt of the iambic familiarization stream used in Experiment 1B; capitalized syllables represent stress**. Middle: segmentation based on transitional probabilities. Bottom: segmentation based on trochaic bias.

The artificial language used in this experiment had the same lexical items, word order, and statistical structure as the language used in Experiment 1A. Two versions of this language were used. In the trochaic language, lexical stress occurred in word-initial position, while in the iambic language lexical stress occurred in word-final position. For an illustration of the competing segmentations indicated by transitional probabilities and lexical stress in the iambic language, see Figure 2.

Lexical stress was created by altering three parameters of the stimuli: pitch contour, amplitude, and duration. The pitch contour in the stressed syllables was based on the pitch contours of an adult native English speaker producing the lexical items. The pitch peak of the vowels varied between 255 and 270 Hz, compared to a monotonic 200 Hz for the unstressed syllables. The pitch contour varied as a function of whether the syllable began with a voiced or a voiceless consonant. For voiced consonants, the pitch contour traced an inverted parabola, peaking near the midpoint of the vowel. For voiceless consonants, the pitch contour began near the peak, and traced a falling plateau. The amplitude of all stressed consonants was increased uniformly by 4 dB. The duration of the stressed syllables was altered by lengthening only the vowels. The average duration of the stressed syllables was 310 ms, compared to 185 ms for unstressed syllables. These languages were identical to those used in Thiessen and Saffran’s (2003) Experiments 1 and 2. The duration of both languages was 140 s. The test items used were identical to those used in Experiment 1A.

#### Procedure

The procedure of this experiment was identical to that used in Experiment 1A.

### Results

If infants segment fluent speech via sensitivity to conditional statistical information, they should show the same pattern of preference in the test phase, regardless of whether they heard the trochaic or iambic language, because the conditional statistical information is identical across these two languages. However, if infants segment the artificial language via lexical stress, they should show the opposite pattern of preference across the two languages, because lexical stress occurs in word-initial position in the trochaic language and word-final position in the iambic language.

**Figure 3:**
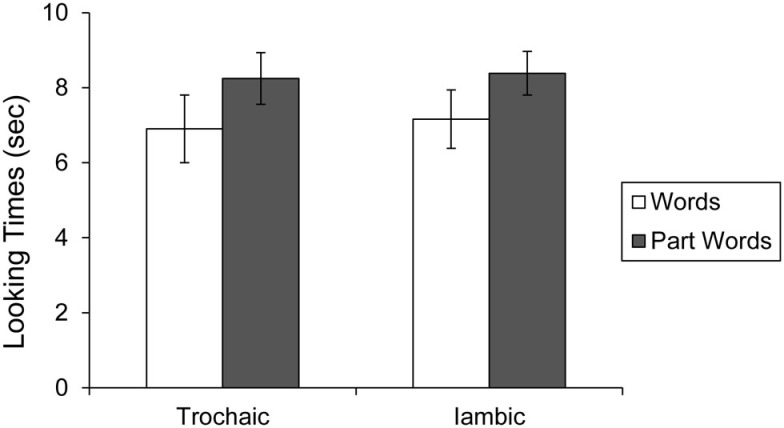
**Looking times to words and part-words in the trochaic and iambic conditions of Experiment 1B**. Error bars indicate standard error.

To determine whether preference for type of test items (words vs. part-words) differed as a function of condition (trochaic vs. iambic language exposure), a 2 × 2 ANOVA (Test Item × Condition) was performed (Figure (3). The main effect for test item (listening to words vs. part-words) was significant, *F*(1, 18) = 11.98, *p* < 0.05, indicating that infants exposed to both languages listened longer to part-words than words. Infants exposed to the trochaic language listened to part-words for 8.25 s (SE = 0.69) and to words for 6.90 s (SE = 0.90). Infants who were exposed to the iambic language listened to part-words for 8.39 s (SE = 0.58) and to words for 7.16 s (SE = 0.78). The main effect of condition (trochaic vs. iambic exposure) was not significant, *F*(1, 18) = 0.041, *p* = 0.84, indicating that infants listened similar lengths of time regardless of which language they heard. The interaction between test item and condition was also not significant, *F*(1, 18) = 0.027, *p* = 0.87, meaning that direction of preference for test items did not differ based on language exposure.

### Discussion

The results of Experiment 1B indicate that, regardless of whether they heard a language made up of trochaic words or iambic words, infants showed the same preference at test. This indicates that infants segmented the same items from both the trochaic and the iambic language. The fact that infants in both groups preferred part-words, as did infants in Experiment 1A, further supports this conclusion. This consistent preference across the trochaic and iambic language indicates that infants segmented the same items from both familiarization streams. The only cue to segmentation that is identical across the streams is the conditional statistical information, indicating that infants segmented on the basis of statistical cues. If infants had relied on lexical stress, they would segment the two languages differently (e.g., Johnson and Jusczyk, 2001; Thiessen and Saffran, 2003).

These results support our prediction that 5-month-olds should rely on conditional statistical information over lexical stress, as do 7-month-olds infants (Thiessen and Saffran, 2003). This is consistent with proposal that use of statistical cues to segment speech develops earlier than use of acoustic cues such as lexical stress. More broadly, this developmental timetable is consistent with the hypothesis that sensitivity to conditional statistical information allows infants to discover a set of lexical forms, which in turn allow infants to identify language-specific acoustic cues such as lexical stress. Rather than statistical cues and acoustic cues being in conflict (as they are artificially placed in the iambic familiarization stream), conditional statistical information may actually allow infants to discover the dominant rhythmic patterns of their native language (Thiessen and Saffran, 2007).

## Experiment 2

Experiments 1A and 1B established that (1) the ability to segment fluent speech on the basis of conditional information is present as early as 5 months of age and (2) that these infants segment on the basis of statistical cues rather than lexical stress cues when they are placed in conflict, replicating the findings of Thiessen and Saffran (2003) with 7-month-olds. By 9 months of age, the weight infants place on conditional statistical cues vis a vis lexical stress has changed, and they rely on stress cues to a greater extent than conditional statistical information. Thiessen and Saffran (2007) suggest that this developmental progression is due to statistical learning. Statistical learning plays two roles in this progression. The first, as demonstrated in Experiment 1, is that infants are able to use conditional statistical information to extract a set of lexical forms from fluent speech. The second is that statistical learning allows infants to identify the commonalities across these word forms, which relies upon distributional (as opposed to conditional) statistical information.

This hypothesis suggests that, once infants have discovered a set of word forms, they integrate information across them. Consider what would happen, for example, if an infant were familiar with the three words *baby*, *diaper*, and *shoe*, and integrated across these word forms. Integrating information across these word forms will emphasize information that is consistent across word forms, while de-emphasizing information that is inconsistent (e.g., Thiessen and Pavlik, 2012). In this case, there is no consistent phonemic information across the three known words, but all three begin with a stressed syllable. Integrating information across a lexicon like this should lead infants to discover that lexical forms can vary in their phonemic identity, but show a consistent word-initial stress pattern. The fact that this pattern is not tied to any particular set of phonemes suggests that it should be widely generalizable, even to new instances. As such, this information could serve to bias subsequent segmentation of novel words. For this hypothesis to be correct, two conditions must be met that would allow infants to learn a lexical stress pattern by 6 months of age (Höhle et al., 2009). First, infants must be able to segment words from fluent speech, via sensitivity to conditional statistical cues, before 6 months. Second, infants must be capable of learning from the distribution of lexical stress in word forms with which they are familiar before 6 months.

Given that Experiment 1 demonstrated that infants are sensitive to conditional statistical regularities in linguistic input, a natural subsequent question to ask is whether infants at this age are also sensitive to distributional statistical regularities in linguistic input. Thus, in Experiment 2, we ask whether 5-month-olds’ learning abilities satisfy the second condition, and they are able to identify a common acoustic feature across lexical forms to which they are exposed. If so, they should be able to discover a prosodic commonality across the word forms to which they are exposed in a laboratory setting. To test this possibility, we exposed 5-month-old infants to lists of trochaic words in isolation and then presented them with either a stream of trochaic or iambic speech. In prior research with 7- and 9-month-old infants, exposure to a list of this kind has been sufficient to allow infants to learn the relation between lexical stress and word position, and to being to use lexical stress as a cue to word segmentation (Thiessen and Saffran, 2007). Note that in prior experiments, English-learning infants have been able to learn both a trochaic and an iambic bias. In this experiment we only exposed infants to a trochaic bias. Previously, we have found that 7- and 9-month-old infants are able to learn an iambic bias that contradicts their native language. Therefore, it is likely that if 5-month-olds – who have less familiarity with the trochaic pattern of English than 7- or 9-month-olds – are able to learn a trochaic pattern, they would also be able to learn an iambic pattern. In this experiment, then, we assess whether 5-month-old infants are able to adapt to the distribution of lexical stress across familiar word forms and acquire a trochaic segmentation bias.

### Materials and methods

#### Participants

Data were obtained from 20 participants between the ages of 5.0 and 5 months, 16 days (*M* = 5.10). Half of these infants were exposed to a trochaic artificial language, and half to an iambic artificial language. To obtain data from 20 infants, it was necessary to run 29 infants. The additional nine infants were excluded (five from the trochaic condition, four from the iambic condition) for crying or squirming during the testing session (4), looking times of less than 3 s to the test trials (3) and experimenter error (2).

#### Stimuli

The trochaic and iambic language, and the test items, used in this experiment were identical to those used in Experiment 1B. Before exposure to the to-be-segmented artificial language, infants heard a list of 30 CVCV bisyllabic nonsense words, repeated twice, for a total of 60 words. Each word in this list was stressed on its first syllable, and there was a pause of 1.4 s between each word; the total length of the 60 word set was 126 s. Lexical stress was created through the alteration of three parameters: pitch contour, amplitude, and duration. The list was identical to that used in Thiessen and Saffran (2007). All of the words in this list were different from the four words that occurred in the familiarization stream.

#### Procedure

The procedure used in this experiment was identical to that used in Experiment 1, with the exception that before the presentation of the to-be-segmented artificial language, infants were exposed to a list of 60 trochaic words (all infants heard the same 60 words), paired with the image of a static checkerboard on the central LCD monitor.

### Results

We compared listening times to words and part-words for infants exposed to both the trochaic language and the iambic language. If infants fail to learn from exposure to a list of trochaic items, they should segment fluent speech – like infants in Experiment 1 – via conditional statistical cues, and show the same pattern of preference after exposure to both the trochaic and iambic segmentation stream. However, if infants learn that lexical stress is a cue to word-initial position, they may begin to use lexical stress as a cue to word segmentation (e.g., Johnson and Jusczyk, 2001). If so, infants should segment different items from the trochaic segmentation stream than from the iambic segmentation stream, and show a different pattern of preference at test after exposure to these two languages.

To assess these possibilities, we performed a 2 (Test item) × 2 (Condition) ANOVA to determine whether infants showed the same, or a significantly different, preference for test items as a function of which segmentation stream they heard. The main effect for test item (listening to words vs. part-words) was not significant, *F*(1, 18) = 0.24, *p* = 0.63, indicating that infants exposed to both languages listened for similar times to words and part-words. The main effect of condition (trochaic vs. iambic exposure) was also not significant, *F*(1, 18) = 0.075, *p* = 0.40, indicating that infants listened for similar lengths of time regardless of which language they heard. However, the interaction between test item and condition was significant, *F*(1, 18) = 17.69, *p* < 0.01, meaning that the direction of preference for test items differed depending on the language to which infants were exposed.

**Figure 4:**
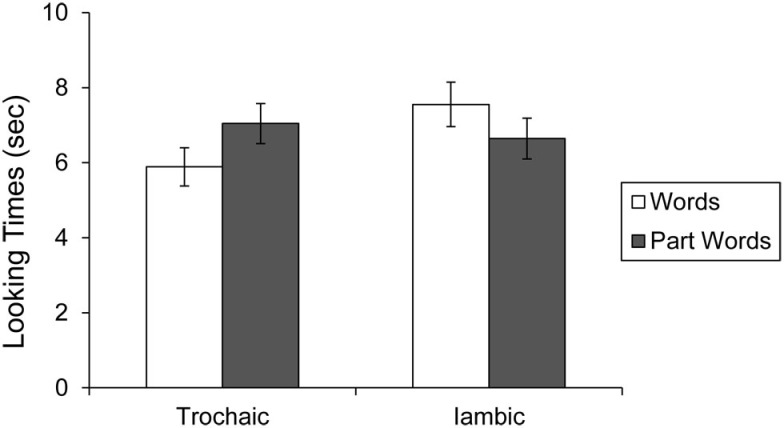
**Looking times to words and part-words in the trochaic and iambic conditions of Experiment 2**. Error bars indicate standard error.

To better understand this interaction, we performed planned *t*-tests comparing listening times to test items in the two conditions. Infants exposed to the trochaic language listened to part-words for 7.04 s (SE = 0.53) and to words for 5.89 s (SE = 0.51). A paired *t*-test revealed that this difference was significant, *t*(9) = 2.93, *p* < 0.05. Infants who were exposed to the iambic language listened to part-words for 6.65 s (SE = 0.54) and to words for 7.55 s (SE = 0.59; See Figure 4). A paired *t*-test revealed that this difference was significant, *t*(9) = 3.12, *p* < 0.05. These results indicate that infants show a different preference for test items after listening to the trochaic and iambic languages, as would be expected if they had learned to treat lexical stress as a cue to word segmentation. Because the placement of lexical stress differs across the two familiarization streams, relying on stress as a cue to segmentation should lead infants to segment different items from them, and therefore prefer different test items.

### Discussion

The fact that infants show a different pattern of preference after listening to the trochaic and iambic familiarization streams indicates that they segmented different items from the two streams. Because the only cue to word boundaries that differs across the two familiarization streams is lexical stress, the different pattern of preference across the two streams indicates that they learned a trochaic lexical stress pattern from exposure to the list of trochaic items, and used this pattern to subsequently segment the fluent speech. This result is consistent with prior experiments demonstrating that infants who rely on lexical stress as a cue to segmentation extract segment words from trochaic input, and actually mis-segment iambic input by treating stressed syllables as word onsets (e.g., Johnson and Jusczyk, 2001; Thiessen and Saffran, 2003). Further, it replicates prior work with 7-month-olds demonstrating that infants – even 5-month-old infants – can learn to use lexical stress as a cue to word segmentation upon exposure to lexical forms that consistently exemplify a stress pattern (Thiessen and Saffran, 2007).

Prior work suggests that 7-month-old English-learning infants are able to learn an iambic stress pattern in addition to the trochaic stress pattern infants learned in this experiment (Thiessen and Saffran, 2007). We did not assess whether the 5-month-olds in this experiment would be able to learn an iambic pattern, which contradicts the predominant pattern of English words. The fact that 7-month-olds can learn such a pattern suggests that 5-month-olds may be able to do so as well, given that 5-month-olds have even less familiarity with the predominant pattern of English to overcome. This is not to suggest that infants at this age are completely unfamiliar with the preferred lexical stress pattern of their native language (e.g., Friederici et al., 2007). To the extent that 5-month-olds are familiar with any lexical items, they have likely already begun to identify some acoustic regularities across those forms. As these results demonstrate, exposure to lexical forms that show a consistent acoustic pattern allows infants to use that consistent information as a cue to subsequent word segmentation, a cue that infants did not rely upon in the absence of such exposure.

## General Discussion

Since the initial demonstration that 8-month-old infants are capable of extracting word forms in fluent speech solely by sensitivity to conditional statistical information, the question of how this sensitivity to statistical information might contribute to language acquisition has been a central one in the field of language development. The current experiments are relevant to that question in two ways. First, they reinforce the claim that sensitivity to statistical information is apparent for linguistic input at a younger age than the commonly cited 7–8 months (c.f. Johnson and Tyler, 2010, for a comparable demonstration with slightly older infants). This suggests that infants may have more opportunity to learn from statistical information than previously thought. Second, they suggest that sensitivity to statistical information can play an important role in helping infants adapt to the acoustic structure of their native language.

One argument against sensitivity to statistical information playing an important role in language acquisition is that real language is more complex than the kinds of artificial stimuli used in laboratory settings, and that statistical learning may not be sufficiently powerful or informative in the face of such complexity (e.g., Johnson and Tyler, 2010). Consistent with this, adults do not weight conditional statistical information very strongly as a cue to word boundaries, instead relying on language-specific segmentation cues (e.g., Mersad and Nazzi, 2011). Similarly, 8- and 9-month-old infants weight language-specific cues, such as lexical stress, more strongly than conditional cues (e.g., Johnson and Jusczyk, 2001; Thiessen and Saffran, 2003). A related argument is that statistical learning develops later than other cues to word segmentation, and is thus not central to the process of language development. For example, some proposals have suggested that the earliest tools for word segmentation are prosodic cues (e.g., Johnson and Jusczyk, 2001; Nazzi et al., 2006). Indeed, German-learning infants have been found to use lexical stress as a cue to word segmentation by 6 months (Höhle et al., 2009), younger than any prior demonstrations that infants were able to use conditional statistical information to segment fluent speech.

The current results present an opportunity to reconsider the relative age at which infants are sensitive to prosodic vs. conditional statistical cues to word segmentation. The 5-month-olds in Experiments 1 and 2 are able to segment words from fluent speech via sensitivity to conditional statistical information, opening the possibility that sensitivity to conditional statistical cues plays a role in learning from a very young age (c.f. Kirkham et al., 2002). Moreover, despite their success at segmenting on the basis of statistical cues, 5-month-old infants do not appear to have developed a trochaic bias. This is consistent with the claim that sensitivity to conditional statistical information develops earlier than sensitivity to language-specific prosodic patterns (Thiessen and Saffran, 2003). Conditional statistical information is potentially available in every linguistic environment, and available without prior knowledge about the acoustic regularities that characterize the language. From our perspective, sensitivity to conditional statistical information is one of a small set of language-universal cues that help infants extract a set of lexical items from the input (for discussion, see Thiessen and Erickson, in press). Once infants have extracted a small set of lexical items, they can begin to learn the language-specific acoustic regularities that will subsequently inform segmentation (Thiessen and Saffran, 2007). If this account is correct, infants would necessarily be able to segment input via conditional statistical information before showing the ability to take advantage of language-specific cues.

This developmental account involves two different aspects of sensitivity to statistical information. Sensitivity to conditional statistical information is one of a small set of language-universal cues that can help infants to extract lexical items from fluent utterances (Thiessen and Erickson, in press). Because items with high conditional probabilities are more likely to be real words in the language than groupings with low conditional probabilities, sensitivity to conditional statistical information helps to guide infants toward discovering a set of lexical items. These lexical items, in turn, are likely to follow the predominant phonological characteristics of the lexical forms in the native language (e.g., Swingley, 2005). This is especially true of the words in infant-directed speech, which appear to exaggerate the regularities present in adult-directed speech (e.g., Fernald and Simon, 1984; Kelly and Martin, 1994).

Once infants have extracted a small set of lexical items from the input, they can learn the phonological regularities that characterize words in the native language. Doing so entails taking advantage of distributional statistical information. Distributional statistical information relates to the frequency and variability of exemplars in the input (e.g., Zhao et al., 2011). It is especially useful in discovering the central tendency or prototypical configuration of some set of exemplars. One linguistically relevant application of this sensitivity to distributional information is category learning. Sensitivity to the frequency of exemplars along a perceptual continuum (e.g., voice onset time) is informative about category boundaries because categories often involve crowds of exemplars near the center of categories, and a sparser group of exemplars at the ambiguous region between categories (e.g., Maye et al., 2002). Sensitivity to variability is similarly informative for category learning; when exposed to distributions with high variability, learners accept a wider range of exemplars as members of the category (e.g., Clayards et al., 2008). A related example of sensitivity to distributional information is the discovery of the prototypical configuration of a set of exemplars. For example, exposure to a set of words allows infants to discover the phonological regularities that characterize those words (Chambers et al., 2003; Saffran and Thiessen, 2003; Thiessen and Saffran, 2007; Thiessen and Yee, 2010).

As these examples illustrates, distributional statistical learning differs from conditional statistical learning in its “output.” Whereas conditional learning results in the segmentation of a discrete item from a larger continuous array of stimuli (such as words from a sentence), distributional learning results in a combination of information from multiple stimuli into a central tendency or prototypical configuration (e.g., Zhao et al., 2011; Thiessen et al., in press). There are several prior models of this kind of information integration, primarily models of long-term memory that combine information across prior instances to identify commonalities (e.g., Hintzman, 1984; McClelland and Rumelhart, 1985). Two of the processes invoked by these models are of particular importance: similarity-based activation, and summation of information across prior instances (for discussion, see Thiessen and Pavlik, 2012). The effect of similarity means that when information is presented, the most similar stored exemplars are most activated and have the greatest influence on the response to the current information. The information in activated memories is then summated, such that information that is consistent across prior activated memories is reinforced, while inconsistent information tends to be canceled out, and an average (weighted toward the most highly activated memories) or prototype can be identified (e.g., Hintzman, 1984). These processes can account for a wide variety of distributional learning phenomena, including category learning, acquired distinctiveness, and the role of variability in facilitating learning of non-adjacent relations (Thiessen and Pavlik, 2012).

Sensitivity to distributional statistical information, achieved by integrating information across many individual exemplars to yield a central tendency, can explain English-learning infants’ acquisition of a trochaic bias. For example, if infants extract the words *BAby*, *DIAper*, and *SHOE*, there is no consistent phonemic information. However, each of the words has a word-initial stress pattern. Integrating across these lexical forms would yield a representation that is not specific to any particular set of phonemes (i.e., is widely generalizable), but strongly indicates that lexical stress is associated with word-initial position. Once infants detect this distributional regularity, it alters their segmentation of subsequent speech (e.g., Thiessen and Saffran, 2007). Experiment 2 demonstrates that even 5-month-olds are capable of this kind of distributional learning. Exposed to a set of lexical items in isolation, 5-month-olds were able to integrate information across these exemplars to identify the only feature consistent across all of them: their lexical stress pattern.

From this perspective, infants’ and adults’ use of phonological cues is not a sign that statistical learning is unimportant for language acquisition. Instead, sensitivity to phonological cues emerges from earlier sensitivity to conditional statistical information in a developmental progression. The cues to which infants are sensitive early in life, such as conditional statistical information or utterance boundaries (e.g., Christophe et al., 2001; Seidl and Johnson, 2006), require no prior experience with or knowledge about a specific language to use. These cues allow infants to discover a set of word forms even before they are familiar with language-specific acoustic cues to word boundaries (e.g., Thiessen and Saffran, 2003). Once infants have discovered a set of words, they can identify language-specific acoustic cues by taking advantage of distributional information about those word forms (Thiessen and Saffran, 2007; Lew-Williams and Saffran, 2012).

The fact that 5-month-old infants are sensitive to both the conditional and distributional regularities necessary to discover a phonological regularity such as lexical stress raises a developmental question: why have 5-month-olds not learned the trochaic pattern of English already? Most prior research indicates that infants do not discover this regularity until some time around 7 months (e.g., Jusczyk et al., 1999; Thiessen and Saffran, 2003). If we are right that discovering such phonological regularities requires infants to first identify a set of lexical items, the lack of a trochaic bias at 5 months likely indicates that infants have yet to become familiar with a sufficient number of words. Even though infants at this age are capable of segmenting fluent speech in a laboratory setting, they may not yet have extracted many words from natural linguistic input. There are several reasons why real languages present a greater challenge than the artificial systems used in experiments like these, including its greater degree of (both inter- and intra-speaker) variability, less robust conditional statistical cues, and a far greater number of lexical items repeated less closely together than in a laboratory setting. These factors may require that infants experience many more repetitions of a word in a natural language to segment it from fluent speech than is necessary in segmentation experiments.

As this discussion indicates, much remains unknown about the exact age at which infants begin to segment words from fluent speech, and the number of lexical forms they are able to extract from fluent native language input (for discussion, see Swingley, 2005). Nevertheless, the current experiments are informative with respect to the relative ordering of the acquisition of different cues to word segmentation. The present studies replicate and extend prior work by Thiessen and Saffran (2003) demonstrating that sensitivity to conditional statistical information in speech is early developing, and appears to emerge – at least in English-learning infants – before the development of the trochaic bias. Though 5-month-olds do not display a trochaic bias, they are able to segment speech via sensitivity to conditional statistical information. Further, they are able to learn a trochaic bias through exposure to a set of words that follow a consistent trochaic pattern. This is also consistent with the hypothesis that segmenting word forms via a domain-general process such as statistical learning is potential mechanism by which infants can develop language-specific acoustic biases (e.g., Thiessen and Saffran, 2007; Thiessen and Erickson, in press).

The ability to segment fluent speech on the basis of the probabilistic relation between sequences of speech sounds is an example of conditional statistical learning. The ability to learn the relation between lexical stress and word position on the basis of a set of exemplars following a particular prosodic pattern is an example of distributional statistical learning. These processes are typically studied and modeled in isolation (e.g., Perruchet and Vinter, 1998; Frank et al., 2010; Thiessen and Pavlik, 2012). But as these experiments indicate, distributional learning constrains subsequent statistical learning, as infants extract items that are consistent with the phonological pattern they have learned. Moreover, we propose that in the course of natural language acquisition, conditional statistical learning influences distributional learning. Infants are able to discover phonological patterns through the lexical forms that they learn via sensitivity to conditional statistical information. To fully understand the role of statistical learning in language acquisition, it will be necessary to develop models and theories that more thoroughly explore how sensitivity to conditional and distributional statistical learning interact to allow infants to adapt to the structure of their native language.

## Conflict of Interest Statement

The authors declare that the research was conducted in the absence of any commercial or financial relationships that could be construed as a potential conflict of interest.
